# Structured triacylglycerol containing behenic and oleic acids suppresses triacylglycerol absorption and prevents obesity in rats

**DOI:** 10.1186/1476-511X-9-77

**Published:** 2010-07-24

**Authors:** Makiko Kojima, Nobuhiko Tachibana, Takashi Yamahira, Satoshi Seino, Ayako Izumisawa, Nobuo Sagi, Toshiharu Arishima, Mitsutaka Kohno, Kiyoharu Takamatsu, Motohiko Hirotsuka, Ikuo Ikeda

**Affiliations:** 1 Food Science Research Institute, Fuji Oil Co., Ltd., 4-3 Kinunodai, Tsukubamirai-shi, Ibaraki 300-2497, Japan; 2 Food Science Research Institute, Fuji Oil Co., Ltd., 1 Sumiyoshi-cho, Izumisano-shi, Osaka 598-8540, Japan; 3 Laboratory of Food and Biomolecular Science, Department of Food Function and Health, Graduate School of Agricultural Science, Tohoku University, 1-1 Tsutsumidori-Amamiyamachi, Aoba-ku, Sendai, Miyagi 981-8555, Japan; 4 Oils & Fats Development Department, Fuji Oil Co., Ltd., 1 Sumiyoshi-cho, Izumisano-shi, Osaka 598-8540, Japan

## Abstract

**Background:**

Dietary 1(3)-behenoyl-2,3(1)-dioleoyl-*rac*-glycerol (BOO) has been reported to inhibit pancreatic lipase activity *in vitro *and suppress postprandial hypertriacylglycerolemia in humans. In the present study, the anti-obesity activities of BOO and its inhibitory effects on lymphatic triacylglycerol (TAG) absorption were investigated in rats.

**Methods:**

In Experiment 1, rats were fed either BOO or soybean oil (SO) diet for 6 weeks. In the BOO diet, 20% of SO was replaced with an experimental oil rich in BOO. In Experiments 2 and 3, rats cannulated in the thoracic duct were administered an emulsions containing trioleoylglycerol (OOO) or an oil mixture (OOO:BOO, 9:1). Tri[1-^14^C]oleoylglycerol (^14^C-OOO) was added to the emulsions administered in Experiment 3.

**Results:**

No observable differences were detected in food intake or body weight gain between the BOO and SO groups in Experiment 1. Plasma and liver TAG concentrations and visceral fat weights were significantly lower in the BOO group than in the SO group. The apparent absorption rate of fat was significantly lower in the BOO group than in the SO group. In Experiment 2, the lymphatic recovery of oleic and behenic acids was significantly lower at 5 and 6 h after BOO administration than after OOO administration. In Experiment 3, the lymphatic recovery of ^14^C-OOO was significantly lower at 5 and 6 h after BOO administration than after OOO administration.

**Conclusions:**

These results suggest that BOO prevents deposition of visceral fat and hepatic TAG by lowering and delaying intestinal absorption of TAG.

## Background

Behenic acid is a long-chain saturated fatty acid consisting of 22 carbon atoms. The physiological functions of structured triacylglycerols (TAGs) containing behenic and medium-chain fatty acids have been examined in previous studies [1-3]. In these studies, structured TAGs were shown to reduce fat absorption in rats. This reduction has been attributed to the low absorbability of behenic acid. Therefore, behenic acid can be utilized as a functional component of low-calorie fats. Dietary fats containing behenic acid are expected to reduce visceral fat deposition. However, only few studies have examined the effects of TAG containing behenic acid on visceral fat deposition.

1(3)-Behenoyl-2,3(1)-dioleoyl-*rac*-glycerol (BOO) is a structured TAG with behenic acid at the 1 or 3 position. In our previous study, analysis of structured TAGs that are resistant to hydrolysis by pancreatic lipase revealed that BOO has an inhibitory effect on this hydrolysis [4]. In addition, Arishima et al. demonstrated that when 10% of dietary fat is replaced with BOO, postprandial serum TAG levels are suppressed in mildly hypertriacylglycerolemic patients [5]. These observations suggest that BOO suppresses intestinal absorption of dietary fat. Several studies have suggested that improvement of postprandial hypertriacylglycerolemia is responsible for the reductions in body fat mass that have been observed in rodents and humans [6-13]. It has also been shown that 1,3-diacylglycerol, oolong tea polymerized polyphenols and tea catechins suppress postprandial hypertriacylglycerolemia by delaying intestinal TAG absorption, in addition to reducing visceral fat deposition [6-9,11]. Therefore, it is believed that BOO has the ability to interfere in fat absorption and prevent visceral fat deposition. In this study, the effects of BOO on visceral fat deposition in rats and fat absorption in lymph-cannulated rats were examined.

## Materials and methods

### Materials

Behenic acid ethyl ester was prepared by heating behenic acid (95% purity; Tokyo Chemical Industry, Tokyo, Japan) that had been solubilized in ethanol containing sulfuric acid (98% purity; Kishida Chemical, Osaka, Japan) as a catalyst. A BOO-rich experimental oil was synthesized by enzymatic interesterification of sunflower oil containing 80% oleic acid (Fuji Oil Co., Osaka, Japan) and behenic acid ethyl ester using 1,3-specific lipase from *Rhizopus niveus*. After elimination of fatty acid ethyl esters by distillation, the residual oil was mixed with n-hexane. Then, the oil/solvent mixture was cooled and crystallized at -5°C. The BOO-rich experimental oil was obtained by filtration followed by evaporation of n-hexane. The percentage of BOO was 41.8% in the experimental oil, which was added to the feed used in Experiment 1. The composition of fatty acids in the experimental oil rich in BOO was as follows: 16:0, 2.6%; 18:0, 3.9%; 18:1n-9, 56.3%; 18:2n-6, 2.9%; 20:0, 3.0% and 22:0, 30.9%. The molecular species of TAG constituting the oil are shown in Table 1, and were analyzed by high performance liquid chromatography (HPLC) (Shimadzu, Kyoto, Japan) using the LiChrosorb RP-18-5 column (5-μm particle, 4.6 mm × 250 mm; GL Sciences, Tokyo, Japan); acetone/acetonitrile solvent, 80/20 (v/v); 2% (v/v) acetone solution concentration; 0.9 ml/min carrier velocity; 20°C and a refractive index detector (Shimadzu). Purified BOO was obtained from the experimental oil by preparative HPLC (Waters, Milford, MA, USA) using the SunFire C18 column (5-μm particle, 50 mm × 250 mm; Waters); acetone/acetonitrile solvent, 80/20 (v/v); 10% (v/v) acetone solution concentration; 1.1 ml/min carrier velocity; 20°C and a refractive index detector (Waters).

**Table 1:** Molecular TAG species in the experimental oil used in Experiment 1

Ingredient	(%)	Species	(%)
St-O-O	56.7		
		BOO	41.8
		SOO	4.3
		POO	1.7
		AOO	3.6
		BLiO	5.3

St-O-St	25.3		
		BOB	6.5
		BOS	4.8
		POS	4.3
		BOP	3.8

OOO	9.4		
DG	5.9		
unknown	2.7		

St: saturated fatty acid; O: oleic acid; B: behenic acid; A: arachidic acid;

S: stearic acid; P: palmitic acid; G: lignoceric acid; DG: diacylglycerol.

### Effect of dietary BOO on visceral fat deposition in rats fed a high-fat diet (Experiment 1)

Six-week-old male Wistar rats were purchased from Japan SLC, Inc. (Shizuoka, Japan). The animals were individually housed under controlled room temperature (23°C ± 1°C), humidity (55% ± 5%) and light-dark-cycle (light from 0700-1900 h). All rats were allowed free access to a commercial chow (CRF-1; Oriental Yeast, Tokyo, Japan) for 4 days. They were divided into 2 groups and fed the assigned experimental diet for 6 weeks. The composition of the experimental diets was as follows (per 100 g diet): 20.0 g casein; 0.3 g L-cystine; 10.0 g sucrose; 18.75 g β-cornstarch; 13.2 g α-cornstarch; 3.5 g AIN-93G mineral mixture (Oriental Yeast); 1.0 g AIN-93 vitamin mixture (Oriental Yeast); 5.0 g cellulose; 0.25 g choline bitartrate and 0.0014 g *tert*-butylhydroquinone. The dietary fat consisted of soybean oil (SO) in the SO group, which was added at a concentration of 28 g per 100 g diet. In the BOO group, 20% of SO was replaced with the BOO-rich experimental oil. As a result, the percentages of BOO and behenic acid in the experimental diets were 2.3% and 1.7%, respectively.

At the end of the feeding period, the rats were fasted for 18 h (1800-1000), following which blood was withdrawn from the abdominal aorta using a heparinized syringe under isoflurane anesthesia. Liver and abdominal adipose tissue samples from epididymal, perirenal and mesenteric sites were carefully removed and weighed. Plasma was separated by centrifugation at 1,900 g for 15 min at 4°C and frozen at -80°C until analyzed.

Feces were collected for 3 days before the end of the feeding period and dried. The total fat content of the feces was calculated by weighing the feces before and after fat extraction using a solvent mixture of petroleum ether and acetic acid (100:1). Plasma TAG and total cholesterol concentrations and aspartate aminotransferase (AST) and alanine aminotransferase (ALT) activities were enzymatically measured using Dry Chem 7000V (FUJIFILM Medical, Tokyo, Japan). Liver lipids were extracted using the procedure described by Folch et al. [14]. TAG and total cholesterol concentrations were measured using previously described methods [15].

### Effect of BOO on lymphatic recovery of fatty acids and tri[1-^14^C]oleoylglycerol in thoracic duct-cannulated rats (Experiments 2 and 3)

Eight-week-old male SD rats were obtained from CLEA Japan (Tokyo, Japan). After a 4-day adaptation period, a cannula was inserted into the left thoracic duct to collect lymphatic fluid and a cannula was inserted into the stomach, using previously described procedures [6]. After surgery, a physiological solution containing 139 mM glucose and 85 mM NaCl was continuously infused overnight at a rate of 3.4 ml/h through the stomach cannula. In Experiment 2, emulsions containing 200 mg trioleoylglycerol (OOO group; Sigma, MO, USA) or an oil mixture of OOO:BOO (9:1) (BOO group), 50 mg fatty acid-free albumin (Wako Pure Chemical Industries, Osaka, Japan) and 200 mg sodium taurocholate (Nacalai Tesque, Kyoto, Japan) were prepared by ultrasonication. After lymph was collected for 2 h (-2 to 0 h), the emulsion was administered to the stomach and the infusion of the glucose/NaCl solution was continued. Lymph was collected at the following intervals after administration of the test emulsions: 0-1, 1-2, 2-3, 3-4, 4-5, 5-6 and 6-8 h. The collection tubes contained EDTA-2Na as an anticoagulant. Lipids were extracted using a chloroform/methanol mixture (2:1, v/v) according to the method described by Folch et al. [14], and then transmethylated using a BF_3_-methanol complex using the method described by Ikeda et al. [16]. Fatty acid methyl esters were analyzed using a SHIMADZU GC-2014 capillary gas-liquid chromatography apparatus equipped with ULBON HR-SS-10 (GL Sciences). Pentadecanoic acid (Alfa Aesar, MA, USA) was used as the internal standard. The fatty acid recovery rate was calculated by subtracting the amount of fatty acids recovered from the lymph collected 2 h prior to the administration of the test emulsion (-2 to 0 h) from the amount of fatty acids recovered from the lymph collected after administration of the test emulsion.

In Experiment 3, thoracic duct-cannulated rats received intragastric administrations of 3 ml of a test emulsion containing 1 μCi of tri[1-^14^C]oleoylglycerol (^14^C-OOO) (PerkinElmer, MA, USA). The composition of the emulsion was the same as that used in Experiment 2, except for the addition of radiolabelled TAG. After administration, lymph was collected for 24 h and radioactivity was measured using a liquid scintillation counter. The maintenance of the experimental rats and all other procedures were identical to those used in Experiment 2.

All rat studies were carried out under procedures consistent with the guidelines for conducting animal experiments prepared by the Graduate School of Agricultural Science at Tohoku University and the Japanese Society for Nutrition and Food Science (Law no. 105 and Notification no. 6 of the Japanese government).

### Statistical analysis

All values are expressed as mean ± SE. Statistical analyses of data was performed using Student's *t*-test. Differences were considered significant at *P *< 0.05 (SPSS Inc., Tokyo, Japan).

## Results

### Effect of dietary BOO on visceral fat deposition in rats fed a high-fat diet (Experiment 1)

No significant differences were observed in food intake or body weight gain between the SO and BOO groups (Table 2). The relative weights of epididymal and mesenteric adipose tissues and liver were significantly lower in the BOO group than in the SO group. Plasma TAG and total cholesterol concentrations were significantly lower in the BOO group than in the SO group (Table 2). Hepatic TAG and cholesterol concentrations were also significantly lower in the BOO group than in the SO group. No significant difference was observed in the concentration of liver phospholipids (data not shown). The activities of plasma AST and ALT were not significantly different between the SO and BOO groups (data not shown). Fecal weight was significantly higher in rats fed the BOO diet than in those fed the SO diet (Table 3). Diarrhea was not observed in either group. Fecal excretion of fatty acids was significantly higher in rats fed the BOO diet than in those fed the SO diet. Both behenic acid and fatty acids other than behenic acid were higher in the BOO group than in the SO group. The apparent absorption rate of fat was significantly lower in rats fed the BOO diet than in those fed the SO diet.

**Table 2:** Body weights, food intakes, visceral fat weights and concentrations of lipid parameters in plasma and liver

	SO	BOO
Initial body weight (g)	160 ± 1	160 ± 1
Final body weight (g)	316 ± 4	309 ± 4
Body weight gain (g/day)	3.7 ± 0.1	3.6 ± 0.1
Food intake (g/day)	12.9 ± 0.2	13.0 ± 0.2

Liver (g/100 g body weight)	2.60 ± 0.02	2.52 ± 0.03*
Adipose tissue weight (g/100 g body weight)		
Epididymal	2.84 ± 0.10	2.49 ± 0.09*
Perirenal	2.75 ± 0.11	2.52 ± 0.12
Mesenteric	1.84 ± 0.06	1.62 ± 0.08*
Total	7.43 ± 0.27	6.62 ± 0.27*

Plasma		
Triacylglycerol (mg/dl)	82.5 ± 7.1	65.1 ± 3.8*
Total cholesterol (mg/dl)	49.3 ± 2.4	42.6 ± 1.9*
Liver		
Triacylglycerol (mg/g Liver)	28.7 ± 3.1	20.9 ± 1.8*
Total cholesterol (mg/g Liver)	5.26 ± 0.19	4.44 ± 0.15*

Values are mean ± SE, n = 14.

* indicates significant differences in corresponding values in rats fed the SO diet (*P *< 0.05).

**Table 3:** Fecal weights and fat excretions in feces

	SO	BOO
Fecal weight (g/day)	0.99 ± 0.02	1.23 ± 0.03*
Fecal fatty acids (mg/day)	88.2 ± 4.7	278 ± 12*
behenic acid (mg/day)	5.96 ± 0.18	125 ± 4*
fatty acids other than behenic acid (mg/day)	82.2 ± 4.6	152 ± 9*
Apparent absorption of fat (%)	97.5 ± 0.1	92.1 ± 0.2*

Values are mean ± SE, n = 14.

* indicates significant differences in corresponding values in rats fed the SO diet (*P *< 0.05).

### Effect of BOO on lymphatic recovery of fatty acids in thoracic duct-cannulated rats (Experiment 2)

**Figure 1:**
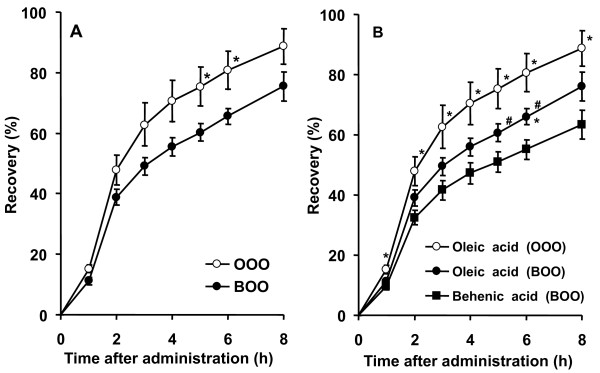
**Effect of BOO on lymphatic recovery of oleic and behenic acids (A) and each fatty acid (B) in rats receiving intragastric administration of fat emulsions**. Values are mean ± SE; n = 5 (OOO group), 7 (BOO group). (A) * indicates significant differences in corresponding values in rats that were administered the BOO emulsion (*P *< 0.05). (B) * indicates significant differences in corresponding values of behenic acid absorption in the BOO group (*P *< 0.05). # indicates significant differences in corresponding values of oleic acid absorption in the OOO group (*P *< 0.05).

No significant differences were observed in the lymphatic flow rates between the OOO and BOO groups (data not shown). The lymphatic recovery rates of oleic and behenic acids are shown in Figure 1A. The lymphatic recovery rates were significantly lower in the BOO group than in the OOO group at 5 and 6 h after administration. The same tendency was observed at 1, 2, 3 and 4 h after administration. The lymphatic recovery rate of oleic acid in the BOO group was also significantly lower compared to that in the OOO group at 5 and 6 h after administration (Figure 1B). The lymphatic recovery rate of the BOO group for behenic acid was significantly lower than that of the OOO group for oleic acid at all time points and in the BOO group at 6 h after administration (Figure 1B).

### Effect of BOO on lymphatic recovery of ^14^C-OOO in thoracic duct-cannulated rats (Experiment 3)

**Figure 2:**
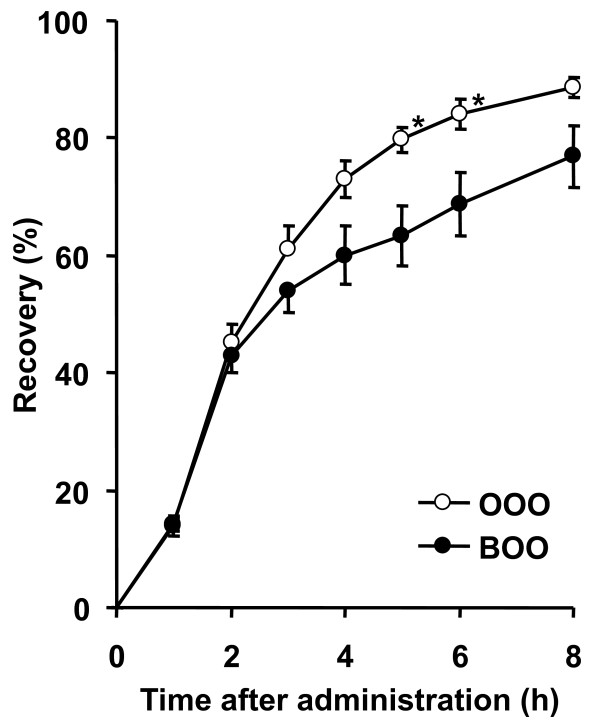
**Effect of BOO on lymphatic recovery of ^14^C-OOO in rats receiving intragastric administration of fat emulsions**. Values are mean ± SE; n = 5 (OOO group), 6 (BOO group). * indicates significant differences in corresponding values of ^14^C-OOO absorption in rats that were administered the BOO emulsion (*P *< 0.05).

No significant differences were observed in the periodic and total lymphatic flow rates between the OOO and BOO groups (data not shown). As shown in Figure 2, the lymphatic recovery of ^14^C-OOO was significantly lower in the BOO group than in the OOO group at 5 and 6 h after administration.

## Discussion

In this study, BOO-induced reduction of visceral fat deposition was demonstrated for the first time (Table 2). Significant reductions in plasma and hepatic TAG concentrations were also observed in the BOO group (Table 2). Fecal excretions of fatty acids, behenic acid and fatty acids other than behenic acid were higher in the BOO group than in the SO group (Table 3). These results suggest that a reduction in fat absorption by dietary BOO causes decreases in plasma and hepatic TAG levels and visceral fat deposition. In the experiment on lymph-cannulated rats, the administration of BOO and OOO reduced the lymphatic recovery rate of oleic acid (Figure 1A). The lymphatic recovery rate of behenic acid was also lower than that of oleic acid (Figure 1B). It was also demonstrated that BOO reduces the lymphatic recovery rate of ^14^C-OOO (Figure 2). These results suggest that the digestion and absorption of BOO and OOO were suppressed in the BOO group. Arishima et al. [4] reported in an *in vitro *study that compared with the administration of only OOO, the administration of an oil mixture of OOO:BOO (9:1) suppresses the hydrolysis of BOO and OOO by pancreatic lipase. This observation suggests that the reduction of TAG absorption is induced by the inhibition of pancreatic lipase in the intestinal lumen. In studies on the absorption of structured TAGs containing behenic and medium-chain fatty acids, low absorbability was ascribed only to behenic acid [1-3]. The present and previous studies [4] have indicated the strong possibility that BOO decreases fat absorption by suppressing the hydrolysis of BOO itself and by inhibiting the hydrolysis of TAGs other than BOO.

Yoshida et al. reported that the apparent absorption of TAG was 39% in rats fed a diet of 2% corn oil and 18% behenic/caproic/caproic TAGs (B66) for 1 week, which was lower than the apparent absorption of TAG in rats fed SO exclusively [1]. The apparent absorption of behenic acid was only 8% [1]. Webb et al. showed that when caprenin containing caprylic, capric and behenic acids was fed to rats, behenic acid absorption was 19% [3]. Based on these observations, they concluded that behenic acid has low bioavailability and its poor absorbability causes reductions in body weight and visceral fat deposition. In the present study, the intake of BOO significantly reduced apparent fat absorption compared with the intake of SO (Table 3). However, compared with the results of previous studies, apparent fat absorption was higher in this study and behenic acid absorption was also obviously higher when rats were fed BOO. This may be due to differences in the behenic acid content of the dietary fats used. In this study, the behenic acid content was 1.7% of the experimental diet, whereas in previous studies, the behenic acid content was 6.8% [1] and 6.1% [3]. It has been previously reported that the lymphatic absorption rate of stearic acid, a fatty acid with a high melting point, when administered as a mixture of oils from completely hydrogenated tallow and soybean oil in various proportions, was inversely proportional to the stearic acid content of the mixed oils [17]. In this study, although the behenic acid content of the administered diets was relatively lower than that used in previous studies, visceral fat amounts were shown to clearly decrease in the BOO group. These results suggest that BOO has the potential to exert an inhibitory effect on fat absorption at relatively low doses. Further studies are necessary to determine the effective dose in humans.

Arishima et al. reported that dietary BOO suppresses postprandial hypertriacylglycerolemia in mildly hypertriacylglycerolemic patients when 10% of dietary fat is replaced with BOO [5]. The present study suggests that this suppression is caused by lower absorption of dietary fat and BOO *via *the inhibition of pancreatic lipase. Since postprandial hypertriacylglycerolemia is thought to be a risk factor for coronary heart disease [18-20], it is possible that BOO has other beneficial effects on the prevention of coronary heart disease, in addition to preventing obesity and dyslipidemia.

The present study demonstrates the possibility that BOO can be utilized as a fat substitute that does not result in an increase in calorie assimilation. BOO might also have some beneficial effects on the prevention of obesity and atherosclerosis through the suppression of postprandial hypertriacylglycerolemia. The melting point of BOO is 31°C, and its use as a supplement to vegetable oil in relatively low amounts would be convenient for use in cooking oils. Dietary BOO did not influence plasma AST and ALT activities in these feeding studies. Therefore, vegetable oils containing BOO can be used as safe and functional cooking oils.

## Conclusions

The present study demonstrated that BOO reduces visceral fat deposition and plasma and hepatic TAG levels in rats. These observations suggest that these reductions are caused by lowering and delaying intestinal absorption of TAG. BOO can be effective for the prevention of obesity and coronary heart disease.

## List of abbreviations used

BOO: 1(3)-behenoyl-2,3(1)-dioleoyl-*rac*-glycerol; SO: soybean oil; TAG: triacylglycerol; OOO: trioleoylglycerol; AST: aspartate aminotransferase; ALT: alanine aminotransferase.

## Competing interests

The authors declare that they have no competing interests.

## Authors' contributions

MK contributed to planning, analysis and manuscript preparation. NT contributed to planning, analysis and interpretation of results. TY, SS and AI contributed to the experimental work. NS contributed to the preparation of the experimental oil. TA contributed to the preparation of the experimental oil and organization of this study. MK, KT and MH contributed to planning and interpretation of the results. II contributed to planning, analysis, interpretation of the results and manuscript preparation. All authors have read and approved the final manuscript.
